# Valorization potential of Egyptian mango kernel waste product as analyzed via GC/MS metabolites profiling from different cultivars and geographical origins

**DOI:** 10.1038/s41598-024-53379-4

**Published:** 2024-02-05

**Authors:** Rehan M. El-Shabasy, Tarek F. Eissa, Yossef Emam, Ahmed Zayed, Nesrin Fayek, Mohamed A. Farag

**Affiliations:** 1 Chemistry Department, Faculty of Science, Menofia University, Shebin El-Kom, 32512 Egypt; 2 Faculty of Biotechnology, October University for Modern Sciences and Arts (MSA), Giza, 12451 Egypt; 3 Pharmacognosy Department, College of Pharmacy, Cairo University, Kasr El Aini St., P.B. 11562, Cairo, Egypt; 4 Pharmacognosy Department, College of Pharmacy, Tanta University, Elguish Street (Medical Campus), Tanta, 31527 Egypt

**Keywords:** Secondary metabolism, Analytical chemistry, Cheminformatics

## Abstract

Increasing attention has been given to mango (*Mangifera indica*) fruits owing to their characteristic taste, and rich nutritional value. Mango kernels are typically discarded as a major waste product in mango industry, though of potential economic value. The present study aims to outline the first comparison of different mango kernel cvs. originated from different localities alongside Egypt, e.g., Sharqia, Suez, Ismailia, and Giza. Gas chromatography–mass spectroscopy (GC–MS) post silylation analysis revealed that sugars were the major class being detected at 3.5–290.9 µg/mg, with some kernels originating from Sharqia province being the richest amongst other cvs. In consistency with sugar results, sugar alcohols predominated in Sharqia cvs. at 1.3–38.1 µg/mg represented by ribitol, iditol, pinitol, and myo-inositol. No major variation was observed in the fatty acids profile either based on cv. type or localities, with butyl caprylate as a major component in most cvs. identified for the first time in mango. Regarding phenolics, Sedeeq cv. represented the highest level at 18.3 µg/mg and showing distinct variation among cvs. posing phenolics as better classification markers than sugars. Multivariate data analyses (MVA) confirmed that the premium cvs “Aweis and Fons” were less enriched in sugars, i.e., fructose, talose, and glucose compared to the other cvs. Moreover, MVA of Zabdeya cv. collected from three localities revealed clear segregation to be chemically distinct. Sharqia originated mango kernels were rich in sugars (e.g., glucose and fructose), whilst sarcosine esters predominated in other origins.

## Introduction

Mango (*Mangifera indica*) is a widely popular tropical fruit belonging to Anacardiaceae family. It is known as the king of fruits owing for its delicious taste, fragrance and potential nutritional value^1^. Mango fruit varies in shape, size, flesh and peel color, taste and aroma, that are all basically dependent on cultivar type^2^. Mango increasing economic potential in the global market^3^, is manifested by its high production yield of mango to rank as the predominant tropical fruit in the twenty-first century^4^. Mango ranks the 5th amongst most cultivated fruit crops worldwide owing for its rich nutrient composition and phytochemicals^5^. It is grown in over 90 countries to amount for ca. 50% of tropical fruits produced worldwide^6^. The world production of mango cultivars reached 55.9 million tons in 2019^7^. The majority of mango trees is cultivated in Asia in particular India^8^ as a major exporter to yield 17–23 million tons^9^. Mango has widely emerged in other countries including China, Indonesia, Thailand and Egypt to all account for 80% of the total world production^1^.

As a major fruit crop, it is represented by 1000 cvs., though only few of them are cultivated (30)^1^. Among worldwide sources of mango, Egyptian mango has gained increasing attention due to its remarkable flavor and taste among consumers^10^. In our previous publication, various cvs. have been investigated, specifically their pulp, based on their volatiles and bioactive compounds using solid-phase microextraction coupled with gas chromatography/mass spectroscopy (SPME-GC/MS)^10^ and bioactive secondary metabolites using ultra-performance liquid chromatography (UPLC) coupled to MS (UPLC/MS) and in relation to its antioxidant activity^11^. According to the development of functional foods, mango peel powder has been included in bakery products, jellies and pastas attributed to its potential antioxidant activity and glycemic index, while mango peels extracts were incorporated in co-pigments and lipid peroxidation inhibitors^12^. These effects have yet to be examined for mango kernels though unlikely as no carotenoids are reported in kernels compared to their richness in fruit pulp and peel^13^.

In recent years, several spectroscopic techniques including mostly hyphenated techniques such as gas/liquid-chromatography coupled to mass-spectroscopy (GC/LC–MS) analysed using multivariate data analyses (MVA) have greatly aided in the holistic characterization of metabolome, and further in samples classification in response to different status or phenotypes^11,14^. Multivariate data analyses (MVA) are typically employed in unsupervised mode, such as principal component analysis (PCA), or supervised one exemplified by orthogonal partial least-squares discriminant analysis (OPLS-DA) for visualization of the rich spectral datasets. Both are routinely incorporated for the classification of investigated specimens, and products fingerprinting and authentication for quality control purposes^15^. Such an approach has increasingly been used for quality control of functional foods in the context of determination of freshness, geographical and genotype, processing, and or adulteration detection^11^.

Following the potential economic value of mango fruits in the world market, we have previously reported on the use of MVA for classification of Egyptian mango cvs. Egypt’s total area under mango cultivation reached 130,000 ha with a total production of 766,128 tons regarded as the most important fruit crop cultivated in all Egyptian province^16^. Fourteen cultivars of mango fruits from different localities were subjected to aroma profiling using headspace solid phase microextraction SPME coupled with gas chromatography mass spectroscopy (GC–MS)^10^, and revealing for distinct aroma profile especially for premium mango cvs. such as Awees being enriched in terpenes^10^. More recently, liquid chromatography mass spectrometry and in comparison, to UV fingerprinting were employed for classification of mango fruit cvs. targeting their specialized metabolites in context to different cvs. and or geographical origin in Egypt^11^. A potential classification was observed from both analytical platforms viz. Ultra-performance liquid chromatography-mass spectrometry (UPLC/MS) and UV spectroscopy, revealing for higher phenolic content in premium Aweis cv concurrent with potential antioxidant effect^11^. UPLC/MS led to the identification of 47 peaks belonging to tannins as gallic acid esters, flavonoids, xanthones, phenolic acids and oxylipids, and confirmed from UV/Vis fingerprinting showing absorption patterns mostly attributed to galloylated conjugates and phenolic acids.

During mango fruits processing, kernels represent the main by-products that is typically discarded presenting a major biowaste in mango industry^17^ though rich in several phytochemicals and nutrients. According to mango varieties, kernel accounts for 10–25% of the whole fruit weight^18^. However, more than one million tons of mango kernels are being annually produced presenting valuable product if subjected to valorization practices increasingly adopted in food industry^18^. To maximize valorization practices and identify potential uses of mango wastes, detailed metabolites characterization is still needed^19^.

Mango kernels typically encompass bioactive components such as, carotenoids, phenolics and ascorbic acid, in addition to macronutrients such as proteins (6–13%), lipids (6–16%) including oleic and stearic acids^20^, carbohydrates (58–80%)^20^. Historically, mango`s kernels were traditionally used to treat gastric related ailments in Indian medicine^21^, such as vermifuge, an astringent in diarrhea, hemorrhages, bleeding hemorrhoids and in cases of gastritis^22^. Further, bioassays have revealed that mango kernel extract exhibited potential antimicrobial effect of potential to be used as food preservative^23^, and as a source of natural antioxidant additive in food processing^17^. Mango kernel has been used in the production of mango butter and seed, which are used in functional foods^1^.

From relevant studies, nutritional composition of mango fruit is clearly dependent on the type/variety of the cultivar, the origin and climatic conditions of its production locality, and maturity^13^, and less reported in case of its kernel or leaf as other byproducts. It is thus crucial to compare the chemical metabolites of different cvs. of mango kernels from several varieties and origin to identify best sources for valorization practices based on detailed chemical composition. This study presents the first metabolomics approach using GC/MS for the classification of Egyptian mango`s kernels as one of the major producers of this fruit worldwide. Kernels were collected from trees grown in different regions alongside Egypt where mango typically grows and further represented by different cvs. targeting their primary metabolites composition using GC/MS and analysed using chemometric tools.

## Material and methods

### Samples collection

Fresh ripe Egyptian mango fruits (17 samples) were collected from farms of Sharqia (30.7° N, 31.63° E), Suez (29.58° N, 32.33° E), and Ismailia (30.35° N, 32.16° E) provinces at the east region of the Nile Delta, in addition to at the west region of the Nile Delta at the west bank of the Nile River (Giza), Suppl. Figs. S1 and S2. All samples were cultivated in sandy soil versus Sharqia cvs. that grown in mixed soil; sand and clay. The irrigation methods varied between drip and spray irrigation. Regarding fertilization, fertilizing mango trees that drip irrigate; Primary irrigation: 2 kg ammonium sulfate + 1 kg magnesium sulfate. Second irrigation: 2 kg compound fertilizer 19 + 19 + 19. Third irrigation: 2 kg potassium sulfate + 2 L phosphoric acid. Fourth irrigation: 2 kg calcium nitrate. Foliar spraying: To increase the percentage of nodes at the beginning of flowering, use coltar (exini compound) at a rate of 75 cm^3^/100 L of water. Foliar spraying is carried out at the following rates in the event of symptoms of deficiency of elements on the trees (200 g sulfate + 200 g chelated zinc + 200 g chelated manganese + 100 g copper sulfate + 100 g magnesium + 50 g borax + 250 g urea (to raise the absorption efficiency) 600 L of water.

Specimens were authenticated by Dr. Tarek Eissa, October University for Modern Sciences and Arts and coded according to the cv. type and geographical origin. The first letter in codes denote for origin as such: S, Suez; Q, Sharqia; I, Ismailia & G, Giza, second letter denotes for cv. name, and third letter for seed as shown in Table 1. In addition, the stage of maturity was confirmed by its external firmness and color, which differs according to each cv. The selected fruits were directly peeled, kernel removed from fruits prior to being stored at −20 °C till analysis.

**Table 1:** List of the collected mango specimens including code, location and description, first letter in codes denote for origin as such: S, Suez; Q, Sharqia; I, Ismailia & G, Giza.

Geographical origin	Sample code	Cultivar (cv.)
Suez	SAS	Aweis
	SNS	Naomy
	SZS	Zabdeya
	SKS	Fagr-Klan
	SFS	Fons
Sharqia province	QAS	Aweis
	QZS	Zabdeya
	QFS	Fons
	QSS	Sadr-hamam
	QDS	Dabsh
	QRS	Arnab
	QQS	Sedeeq
	QNS	Nabela
	QGS	Golphia
	QHS	Hindi
Ismailia governorate	IMS	Mazraa
Giza	GZS	Zabdeya

Second letter denotes for cv. name, and third letter for seed.

Voucher specimens are kept in the Pharmacognosy Department, Faculty of Pharmacy, Cairo University with the same codes used in the current studs after the addition of the department’s initials and year of collection. For instance, SAS was kept under the voucher code PG_CU_SAS_2021. All experimental procedures were carried out in accordance with the relevant laws and guidelines, including the appropriate permissions for the collection of plant specimens.

### Samples preparation

Mango’s dried kernel was grounded separately using mortar and pestle under liquid nitrogen. The powdered seed (30 mg) was homogenized with 2.5 mL methanol containing 5 μg/mL xylitol (as internal standard for relative quantification) using a Turrax mixer. To prevent extra heating, homogenization was operated at 11,000 rpm for five 20 s periods, separated by 1 min of recession. After that, extract was vortexed vigorously and centrifuged at 3000*g* for 30 min to remove debris; with 100 μL aliquoted for chemical analysis. Three biological replicates were carried out for each kernel sample^24^.

### GC/MS analysis

Kernel dried extracts (100 μL) were obtained via evaporation under nitrogen stream. About 150 μL of *N*-methyl-*N*-(trimethylsilyl) trifluoroacetamide (MSTFA) was used in derivatization for 45 min at 60 °C. Before GC/MS analysis, sample equilibrium was thoroughly processed via Shimadzu GC-17A gas chromatograph coupled to Shimadzu QP5050A mass spectrometer at 28 °C. The applied column (Rtx-5MS) was described by 30 m of length with inner diameter at 0.25 mm while the thickness film was 0.25 μm. Split mode was implemented for injections with a split ratio of 1:15 under conditions of : injector temp. 280 °C, column oven temp. 80 °C for 2 min, then modified to 315 °C at a rate of 5 °C/min, and kept isothermally at 315 °C for 12 min, when the flow rate of carrier gas (He) was 1 mL/min. Transfer line temp. was set at 280 °C and ion source temp. adjusted at 180 °C. Electron ionization mode (EI, 70 eV) with a scan range of *m/z* 50–650 was used. AMDIS software (https://www.amdis.net) was involved in identification; firstly peaks were deconvoluted to determine the silylated metabolites then comparing their retention indices (RI) with *n*-alkanes series (C8–C40), and mass matching to NIST^25^ and WILEY library databases and with standards if possible according to previously reported procedure^26^.

### Metabolites identification, quantification and modelling

GC–MS files were converted to. netcdf file format using through MS Convert option in Shimadzu program, then to abf files utilizing ABF converter (https://www.reifycs.com/AbfConverter/). In that regard, data analysis was performed using MS dial software (http://prime.psc.riken.jp/compms/msdial/main.html) according to the following parameters: mass range (0–500 Da), MS1 tolerance for alignment (0.015 Da), retention time (0–30 min), minimum peak height (1000), sigma (0.7), accurate mass tolerance (MS) 0.01 Da, and peak height 1000. Alcohols, organic acids, fatty acids, soluble sugars and free amino acids were quantified using standard curves of glycerol, lactic acid, stearic acid, glucose and glycine and expressed as mg/g. For the standard curves, eight serial dilutions were prepared (from 10 to 600 μg/mL) following conditions cited in Fahmy et al.^27^. Peak abundance was exported for multivariate data analysis where final ID and metabolites were Pareto scaled using SIMCA 14.1 (Umetrics, Umea, Sweden) in which the obtained data were subjected to principal component analysis (PCA) and orthogonal partial least squares discriminant analysis (OPLS-DA). PCA was carried out to show the variance of metabolites amongst different samples whilst information on differences in the metabolite composition can be professed by OPLS-DA. In addition, Q^2^ and R^2^ were involved to induce the performance of the chemometric models and number of permutations; Q^2^ reflects the model predictability and R^2^ determine the fit goodness. The cross-validation method Q^2^ applied was the "k-fold cross validation" in which the calibration set is divided into subsets using a sevenfold. The distance to the model (DModX) was calculated to define outliers whereas Hotelling's T2 was utilized for diagnosis of strong outliers for the OPLS-DA plot^24^.

## Results and discussion

The current study aimed to assess kernels´ metabolome heterogeneity in several mango cvs. represented by 17 cvs. cultivated in different regions in Egypt (e.g., Suez, Sharqia, Giza, and Ismailia) (Table 1). Such comprehensive metabolite profile could aid to identify best cvs. enriched in a certain chemical for future valorization purposes. To assess biological variance for each sample and analysis conditions, three biological replicates were analysed under same conditions using GC–MS following silylation by MSTFA. The use of MSTFA is based due its reaction with many labile functional groups commonly found in organic compounds (e.g., hydroxyl group of polar low molecular weight metabolites as sugars and amino groups in amino acids) to form the more volatile non-polar trimethylsilyl (TMS)-ether derivatives. Such derivatization reactions have broadened the scope of GC–MS analysis to other non-volatile compounds, including sugars, amino acids, and fatty acids^28^.

**Figure 1:**
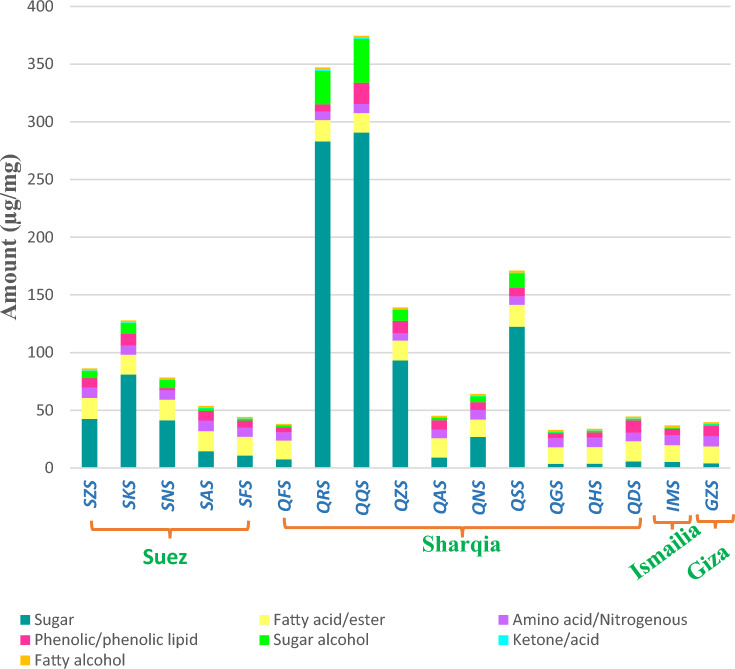
The phytochemical constituents of different cvs. of mangos` kernels originated from four localities in Egypt including Suez, Sharqia, Ismailia and Giza (e.g. SZS, SKS, SNS, SAS, SFS, QFS, QRS, QQS, QZS, QAS, QNS, QSS, QGS, QHS, QDS, IMS, and GZS) expressed in µg/mg. Sugars appear as the major metabolite class followed by fatty acids. For mango codes refer to Table 1.

**Figure 2:**
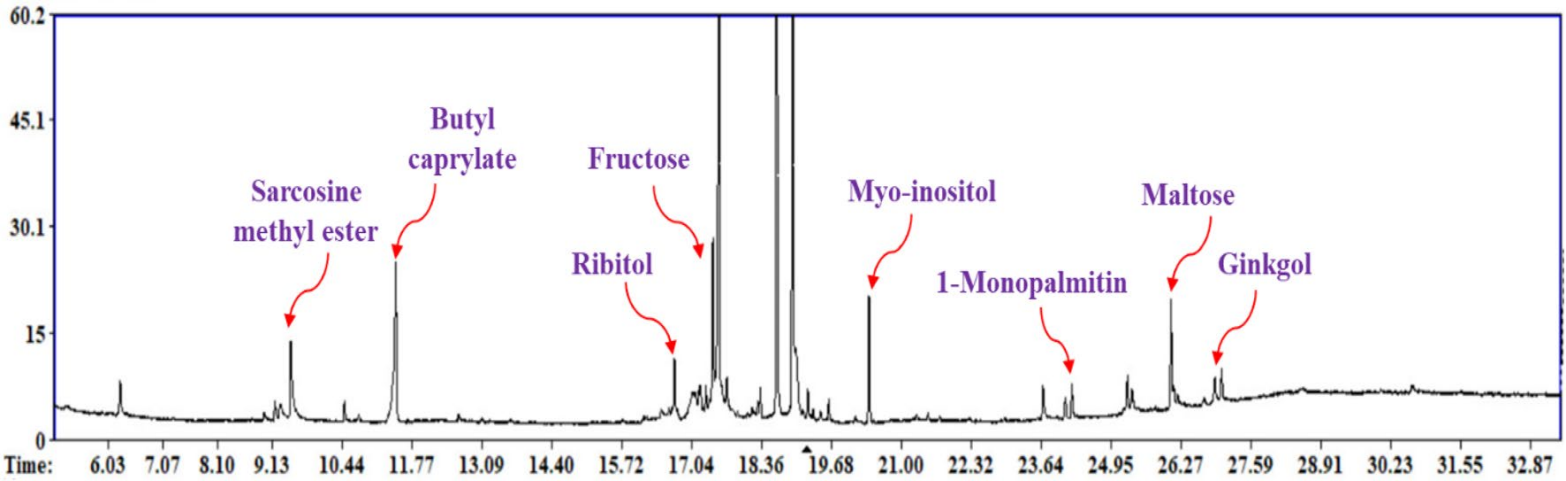
A representative GC–MS chromatogram with the major identified constituents from mango kernel collected from different Egyptian regions.

A total of 41 peaks were identified in mango kernels belonging to different classes including sugars (15), fatty acids/esters (6), amino acids (5), sugar alcohols (6), nitrogenous compounds (5), phenolics and phenolic lipids (4). In addition, trace levels of fatty alcohols, ketones, acids and alcohols were detected as displayed in Fig. 1. GC–MS chromatogram showed the major chemical constituents associated by the most abundant classes in mango kernels (Fig. 2**)**.

Quantitative variance of the identified metabolites in mango kernels were represented in Table 2. The tabulated data revealed the significant abundance of sugars particularly in Seedeq and Arnab cvs. The highest sugar level was detected in cvs. QQS, QRS, and QSS at 290.7, 283 and 122.5 µg/mg compared to GZS and IMS at 4 and 5.2 µg/mg, respectively. Seedeq mango is a creamy fruit with no fibre and a distinct sweet sugary taste and texture that distinguishes it from other varieties, making it suitable for the majority of individuals^29^. Besides, mango has the ability to be devolved into crystalized sugar, which may be considered a great substitute for cane sugar. It is also high in antioxidants and polyphenols, which have health benefits such as improving lipid profiles, stabilizing blood glucose fluctuations, and perhaps acting as an antidiabetic sugar^30^. Hence, QQS and QRS cvs. are highly recommended for further investigation to be involved as antidiabetic sugar due to the high percentage of antioxidants and polyphenols. Interesting to notice that Aweis and Fons cvs. displayed a small change in sugar levels based on origin, being detected at 14.5 µg/mg in SAS compared with QAS at 8.9 µg/mg, while SFS and QFS encompassed 10.8 and 7.6 µg/mg, respectively. In addition, Aweis and Fons were founded to be richer in fatty acids than sugars and this enhanced their test and flavor attributed to abundance of butyl caprylate for the first time in the Egyptian mango. In consistency with sugar results, sugar alcohols were more predominated in both QQS and QRS cvs. Variation of each volatile class will be deeply investigated in the following subsections.

**Table 2:** Quantities of identified metabolites in Mango kernel by GC–MS post silylation expressed as mean µg/mg ± SD (n = 3). The meaning of sample codes is listed in Table 1.

Peak #	Average Rt (min)	Average RI	Metabolite name	Class	SZS	SKS	SNS	SAS	SFS	QFS	QRS	QQS	QZS	QAS	QNS	QSS	QGS	QHS	QDS	IMS	GZS
1	5.924	974.09	1-Penten-3-ol	Alcohols	0.06 ± 0.0	0.05 ± 0.0	0.06 ± 0.0	0.05 ± 0.0	0.05 ± 0.0	0.05 ± 0.0	0.05 ± 0.0	0.04 ± 0.0	0.06 ± 0.0	0.06 ± 0.0	0.04 ± 0.0	0.05 ± 0.0	0.04 ± 0.0	0.04 ± 0.0	0.05 ± 0.0	0.04 ± 0.0	0.04 ± 0.0
3	9.192	1205.48	Sarcosine, *N*-trifluoroacetyl-, ethyl ester	Amino acid/nitrogenous compounds	1.68 ± 0.43	1.08 ± 0.15	1.76 ± 0.45	2.21 ± 0.14	1.48 ± 0.38	1.45 ± 0.05	0.54 ± 0.16	0.46 ± 0.13	0.38 ± 0.29	1.34 ± 0.45	0.87 ± 0.1	0.68 ± 0.12	1.01 ± 0.52	1.05 ± 0.29	0.97 ± 0.43	1.21 ± 0.1	1.57 ± 0.21
4	9.268	1210.53	Sarcosine, *N*-trifluoroacetyl-, methyl ester		1.99 ± 0.26^b,c,d^	1.69 ± 0.19^c,d^	1.67 ± 0.33^c,d^	2.08 ± 0.22^a,b,c,d^	2.14 ± 0.22^a,b,c,d^	1.69 ± 0.21^c,d^	1.03 ± 0.3^d^	1.85 ± 0.4 ^b,c,d^	1.90 ± 0.68^b,c,d^	1.90 ± 0.23^b,c,d^	2.58 ± 0.55^a,b,c^	1.73 ± 0.31^c,d^	3.24 ± 0.35^a^	3.01 ± 0.53^a,b^	2.42 ± 0.53^a,b,c^	2.95 ± 0.68^a,b^	2.97 ± 0.07^a,b^
5	9.492	1225.08	Sarcosine, *N*-trifluoroacetyl-, methyl ester isomer		4.83 ± 0.77	4.57 ± 0.5	4.58 ± 0.38	4.77 ± 0.3	3.96 ± 0.51	3.69 ± 0.21	4.92 ± 0.12	4.87 ± 0.27	3.33 ± 1.27	3.96 ± 0.66	4.69 ± 0.49	4.47 ± 0.7	3.55 ± 0.56	4.14 ± 0.78	4.08 ± 0.85	4.19 ± 1.0	4.41 ± 0.17
6	10.489	1289.91	Nicotinic acid- TMS		0.18 ± 0.11	0.31 ± 0.15	0.18 ± 0.06	0.10 ± 0.04	0.06 ± 0.01	0.06 ± 0.01	0.39 ± 0.15	0.57 ± 0.03	0.39 ± 0.12	0.08 ± 0.04	0.12 ± 0.1	0.22 ± 0.08	0.05 ± 0.01	0.04 ± 0.01	0.05 ± 0.01	0.06 ± 0.03	0.04 ± 0.02
7	10.772	1308.3	l-Threonine-2 TMS		0.09 ± 0.03	0.23 ± 0.18	0.16 ± 0.07	0.08 ± 0.02	0.12 ± 0.08	0.14 ± 0.05	0.19 ± 0.15	0.18 ± 0.13	0.21 ± 0.12	0.07 ± 0.02	0.07 ± 0.04	0.37 ± 0.28	0.04 ± 0.01	0.04 ± 0.02	0.04 ± 0.0	0.04 ± 0.01	0.04 ± 0.01
**Total amino acids/nitrogenous compounds**					**8.83 ± 1.6**	**7.93 ± 1.17**	**8.40 ± 1.29**	**9.29 ± 0.71**	**7.81 ± 1.2**	**7.07 ± 0.53**	**7.12 ± 0.9**	**7.98 ± 0.96**	**6.28 ± 2.49**	**7.41 ± 1.39**	**8.37 ± 1.28**	**7.52 ± 1.49**	**7.93 ± 1.46**	**8.31 ± 1.63**	**7.61 ± 1.83**	**8.49 ± 1.82**	**9.08 ± 0.48**
8	11.472	1353.85	Butyl caprylate	Fatty acids/esters	14.34 ± 0.77	13.26 ± 0.31	14.19 ± 0.39	13.98 ± 0.57	13.32 ± 0.3	13.63 ± 0.48	13.64 ± 0.37	12.22 ± 0.42	13.94 ± 0.25	14.01 ± 0.34	11.99 ± 0.26	14.46 ± 0.18	11.72 ± 0.18	11.79 ± 0.01	14.1 ± 0.93	11.72 ± 0.42	11.89 ± 0.16
23	18.438	1916.53	Methyl palmitate		0.10 ± 0.02	0.09 ± 0.01	0.10 ± 0.01	0.11 ± 0.02	0.10 ±	0.10 ± 0.01	0.11 ± 0.01	0.05 ± 0.06	0.08 ± 0.01	0.11 ± 0.01	0.10 ± 0.01	0.12 ± 0.02	0.10 ± 0.01	0.10 ± 0.01	0.12 ± 0.0	0.13 ± 0.06	0.10 ± 0.01
28	19.645	2033.27	Palmitic acid- TMS		0.39 ± 0.17	0.63 ± 0.1	0.41 ± 0.06	0.28 ± 0.12	0.20 ± 0.06	0.19 ± 0.03	1.24 ± 0.36	1.20 ± 0.18	0.49 ± 0.1	0.24 ± 0.07	0.33 ± 0.11	0.85 ± 0.16	0.10 ± 0.01	0.14 ± 0.02	0.20 ± 0.01	0.11 ± 0.04	0.14 ± 0.02
30	21.266	2200.78	Linoleic acid- TMS		0.02 ± 0.01	0.03 ± 0.0	0.02 ± 0.01	0.02 ± 0.0	0.01 ± 0.0	0.01 ± 0.01	0.11 ± 0.02	0.11 ± 0.0	0.02 ± 0.0	0.02 ± 0.01	0.03 ± 0.01	0.04 ± 0.01	0.01 ± 0.0	0.02 ± 0.01	0.02 ± 0.0	0.01 ± 0.0	0.01 ± 0.0
31	21.515	2228.61	Stearic acid- TMS		0.38 ± 0.05	0.41 ± 0.03	0.22 ± 0.02	0.14 ± 0.06	0.16 ± 0.04	0.08 ± 0.02	0.81 ± 0.4	0.88 ± 0.11	0.35 ± 0.08	0.12 ± 0.01	0.24 ± 0.1	0.73 ± 0.1	0.10 ± 0.01	0.10 ± 0.01	0.12 ± 0.0	0.13 ± 0.06	0.14 ± 0.02
34	24.232	2550.91	1-Monopalmitin- TMS		2.95 ± 0.05	2.68 ± 0.10	2.80 ± 0.08	2.79 ± 0.13	2.39 ± 0.16	2.12 ± 0.06	2.58 ± 0.06	2.46 ± 0.02	2.48 ± 0.21	2.31 ± 0.16	2.38 ± 0.06	2.64 ± 0.13	2.32 ± 0.05	2.30 ± 0.18	2.82 ± 0.07	2.48 ± 0.28	2.32 ± 0.06
**Total fatty acids/esters**					**18.18 ± 1.06**	**17.11 ± 0.56**	**17.76 ± 0.58**	**17.32 ± 0.9**	**16.19 ± 0.57**	**16.15 ± 0.6**	**18.49 ± 1.23**	**16.92 ± 0.8**	**17.36 ± 0.67**	**16.81 ± 0.6**	**15.06 ± 0.56**	**18.84 ± 0.6**	**14.35 ± 0.25**	**14.46 ± 0.23**	**17.38 ± 0.49**	**14.59 ± 0.87**	**14.6 ± ** **0.28**
32	23.69	2482.3	Behenic alcohol	Fatty alcohols	1.30 ± 0.15	1.11 ± 0.05	1.19 ± 0.05	1.23 ± 0.1	1.05 ± 0.09	0.81 ± 0.07	1.28 ± 0.13	1.13 ± 0.04	0.92 ± 0.08	1.00 ± 0.11	1.26 ± 0.0	1.20 ± 0.1	1.27 ± 0.04	1.25 ± 0.07	1.36 ± 0.05	1.37 ± 0.14	1.33 ± 0.1
33	24.116	2536.4	1-Docosanol		0.42 ± 0.01	0.46 ± 0.07	0.46 ± 0.12	0.35 ± 0.15	0.19 ± 0.05	0.02 ± 0.02	0.79 ± 0.14	0.76 ± 0.07	0.33 ± 0.12	0.17 ± 0.03	0.26 ± 0.12	0.56 ± 0.09	0.14 ± 0.0	0.15 ± 0.01	0.17 ± 0.01	0.14 ± 0.03	0.16 ± 0.02
**Total fatty alcohols**					**1.71 ± 0.16**	**1.56 ± 0.12**	**1.65 ± 0.17**	**1.58 ± 0.24**	**1.24 ± 0.14**	**0.83 ± 0.09**	**2.07 ± 0.27**	**1.90 ± 0.11**	**1.25 ± 0.2**	**1.17 ± 0.14**	**1.52 ± 0.15**	**1.77 ± 0.19**	**1.41 ± 0.04**	**1.40 ± 0.08**	**1.53 ± 0.06**	**1.52 ± 0.17**	**1.48 ± 0.12**
2	6.267	1004.97	Cyclohexanone, 3,3,5-trimethyl-	Ketones/acids	0.55 ± 0.03	0.54 ± 0.01	0.54 ± .0.02	0.53 ± 0.03	0.55 ± 0.01	0.56 ± 0.02	0.52 ± 0.02	0.47 ± 0.01	0.59 ± 0.02	0.58 ± 0.02	0.46 ± 0.01	0.56 ± 0.01	0.45 ± 0.01	0.45 ± 0.0	0.55 ± 0.01	0.45 ± 0.02	0.45 ± 0.01
13	17.195	1799.8	Shikimic acid- 4 TMS		0.07 ± 0.03	0.19 ± 0.08	0.10 ± 0.06	0.04 ± 0.03	0.01 ± 0.0	0.01 ± 0.0	0.30 ± 0.07	0.28 ± 0.08	0.12 ± 0.05	0.01 ± 0.01	0.03 ± 0.03	0.10 ± 0.04	0.01 ± 0.01	0.01 ± 0.0	0.01 ± 0.01	0.00 ± 0.0	0.01 ± 0.0
**Total ketones/acids**					**0.62 ± 0.06**	**0.72 ± 0.09**	**0.64 ± 0.08**	**0.57 ± 0.06**	**0.56 ± 0.02**	**0.57 ± 0.02**	**0.82 ± 0.09**	**0.76 ± 0.1**	**0.71 ± 0.06**	**0.60 ± 0.03**	**0.49 ± 0.04**	**0.66 ± 0.06**	**0.46 ± 0.02**	**0.45 ± 0.01**	**0.56 ± 0.02**	**0.46 ± 0.03**	**0.46 ± 0.01**
9	11.844	1377.9	1-(2,3-Dimethoxyphenyl) ethanol	Phenolics/phenolic lipids	0.28 ± 0.15	0.38 ± 0.07	0.01 ± 0.0	0.03 ± 0.03	0.23 ± 0.05	0.10 ± 0.1	1.43 ± 0.53	1.35 ± 0.08	1.04 ± 0.03	0.47 ± 0.29	0.63 ± 0.14	0.80 ± 0.22	0.03 ± 0.01	0.11 ± 0.04	0.18 ± 0.08	0.10 ± 0.03	0.27 ± 0.1
35	25.223	2676.55	Phenol, 3-(eptadecadienyl-		0.14 ± 0.04	0.32 ± 0.09	0.06 ± 0.02	0.06 ± 0.02	0.12 ± 0.03	0.07 ± 0.02	0.23 ± 0.04	1.67 ± 0.63	0.13.03	0.07 ± 0.02	0.08 ± 0.01	0.14 ± 0.04	0.05 ± 0.0	0.04 ± 0.0	0.09 ± 0.01	0.04 ± 0.0	0.07 ± 0.01
37	25.364	2694.59	Phenol, 3-(heptadecenyl)- (Cardanol)		2.27 ± 0.53	5.30 ± 0.48	1.20 ± 0.05	1.84 ± 0.08	5.71 ± 1.02	1.44 ± 0.1	2.49 ± 0.15	13.55 ± 3.33	1.79 ± 0.27	1.87 ± 0.18	2.37 ± 0.16	1.81 ± 0.55	1.27 ± 0.07	0.77 ± 0.03	2.67 ± 0.3	1.51 ± 0.13	1.89 ± 0.13
40	26.927	2865.72	Phenol, 3-(pentadecenyl)- (Ginkgol)		5.70 ± 1.17	4.20 ± 0.44	0.45 ± 0.17	6.47 ± 0.31	0.22 ± 0.09	2.80 ± 0.32	2.34 ± 0.16	1.78 ± 0.16	7.81 ± 1.33	5.86 ± 0.47	3.69 ± 0.43	4.63 ± 0.4	2.80 ± 0.15	3.60 ± 0.13	7.69 ± 0.64	4.02 ± 0.32	6.6 ± 0.04
**Total phenolics/phenolic lipids**					**8.40 ± 1.88**	**10.21 ± 1.08**	**1.72 ± 0.24**	**8.40 ± 0.44**	**6.28 ± 1.18**	**4.41 ± 0.54**	**6.50 ± 0.87**	**18.36 ± 4.21**	**10.78 ± 1.92**	**8.27 ± 0.97**	**6.77 ± 0.73**	**7.38 ± 1.21**	**4.15 ± 0.24**	**4.52 ± 0.2**	**10.63 ± 1.03**	**5.67 ± 0.48**	**8.82 ± 0.68**
11	16.732	1760.67	Ribose-4 TMS	Sugars	0.40 ± 0.18	1.26 ± 0.43	0.48 ± 0. 26	0.18 ± 0.15	0.05 ± 0.02	0.03 ± 0.01	4.13 ± 0.99	3.59 ± 1.14	0.74 ± 0.28	0.05 ± 0.03	0.18 ± 0.2	0.86 ± 0.38	0.02 ± 0.01	0.02 ± 0.01	0.03 ± 0.01	0.03 ± 0.02	0.02 ± 0.0
14	17.327	1812.06	Methyl *α*-arabinofuranoside-3 TMS		0.90 ± 0.2^c^	1.88 ± 0.36^c^	1.07 ± 0.14^c^	0.42 ± 0.25^c^	0.28 ± 0.13^c^	0.22 ± 0.08^c^	15.11 ± 20.7^a^	17.85 ± 4.15^a^	3.50 ± 1.49^b,c^	0.29 ± 0.12^c^	1.68 ± 1.6^c^	7.76 ± 2.8^b^	0.26 ± 0.1^c^	0.18 ± 0.03^c^	0.33 ± 0.17^c^	0.32 ± 0.22^c^	0.15 ± 0.09^c^
15	17.457	1824.39	Fructose-5 TMS		6.32 ± 1.92^c,d^	13.47 ± 4.1^b,c,d^	8.38 ± 2.15^c,d^	2.81 ± 1.62^d^	1.74 ± 0.67^d^	1.79 ± 0.71^d^	55.25 ± 9.43^a^	53.77 ± 10.82^a^	20.56 ± 6.39^b,c^	2.15 ± 0.72^d^	5.84 ± 5.23^d^	22.93 ± 9.11^b^	0.71 ± 0.2^d^	0.60 ± 0.23^d^	0.94 ± 0.41^d^	0.98 ± 0.53^d^	0.48 ± 0.14^d^
17	17.601	1838.97	Unknown monosaccharide-5 TMS		0.04 ± 0.01	0.04 ± 0.0	0.04 ± 0.01	0.02 ± 0.0	0.02 ± 0.0	0.02 ± 0.0	0.07 ± 0.02	0.06 ± 0.01	0.04 ± 0.01	0.03 ± 0.0	0.02 ± 0.0	0.05 ± 0.01	0.01 ± 0.01	0.01 ± 0.0	0.02 ± 0.0	0.02 ± 0.01	0.02 ± 0.01
18	17.724	1849.44	Methyl *α*-d-glucofuranoside-4 TMS		0.67 ± 0.12	1.32 ± 0.28	0.57 ± 0.09	0.28 ± 0.16	0.22 ± 0.08	0.17 ± 0.06	6.87 ± 1.2	7.07 ± 1.36	2.12 ± 0.66	0.19 ± 0.06	0.47 ± 0.36	2.81 ± 0.99	0.09 ± 0.03	0.08 ± 0.03	0.15 ± 0.07	0.12 ± 0.06	0.09 ± 0.01
22	18.354	1908.78	Unknown monosaccharide-5 TMS		1.22 ± 0.25	2.45 ± 0.18	0.86 ± 0.45	0.63 ± 0.34	0.46 ± 0.26	0.30 ± 0.13	9.55 ± 2.17	11.88 ± 2.48	2.58 ± 1.25	0.31 ± 0.02	1.25 ± 0.91	5.30 ± 1.34	0.12 ± 0.0	0.14 ± 0.04	0.24 ± 0.05	0.19 ± 0.05	0.16 ± 0.04
24	18.661	1937.72	Talose-5 TMS		13.70 ± 3.57^b,c,d,e^	33.93 ± 10.08^b,c,d^	12.65 ± 4.41^e^	6.63 ± 3.97^e^	3.99 ± 1.5^e^	2.54 ± 0.96^e^	97.08 ± 19.03^a^	100.15 ± 19.76^a^	38.92 ± 11.19^b^	2.96 ± 1.26^e^	8.20 ± 6.4^d,e^	38.24 ± 13.27^b,c^	1.04 ± 0.32^e^	1.08 ± 0.39^e^	1.85 ± 0.78^e^	1.60 ± 0.8^e^	0.76 ± 0.25^e^
25	19.023	1972.13	Glucose-4 TMS		7.85 ± 1.49^c^	14.62 ± 2.9^c^	6.69 ± 0.98^c^	2.81 ± 1.42^c^	2.63 ± 1.2^c^	2.00 ± 0.7^c^	86.63 ± 16.59^a^	94.38 ± 19^a^	21.35 ± 6.75^b,c^	2.13 ± 0.34^c^	8.52 ± 6.36^c^	38.68 ± 13.5^b^	1.03 ± 0.26^c^	1.19 ± 0.32^c^	1.87 ± 0.51^c^	1.69 ± 0.68^c^	1.32 ± 0.34^c^
26	19.249	1993.08	Unknown monosaccharide-5 TMS		0.33 ± 0.08	1.00 ± 0.32	0.27 ± 0.16	0.29 ± 0.26	0.06 ± 0.02	0.04 ± 0.01	0.55 ± 0.17	0.56 ± 0.24	0.38 ± 0.19	0.04 ± 0.01	0.06 ± 0.05	0.20 ± 0.13	0.01 ± 0.0	0.01 ± 0.0	0.01 ± 0.0	0.01 ± 0.0	0.01 ± 0.0
36	25.275	2682.95	Unknown disaccharide- TMS		0.45 ± 0.27	0.99 ± 0.86	0.68 ± 0.53	0.09 ± 0.09	0.08 ± 0.09	0.06 ± 0.05	0.12 ± 0.04	0.48 ± 0.20	0.07 ± 0.01	0.04 ± 0.01	0.07 ± 0.09	0.06 ± 0.02	0.06 ± 0.04	0.06 ± 0.08	0.03 ± 0.02	0.07 ± 0.04	0.14 ± 0.03
38	25.712	2738.47	Sucrose- TMS		0.07 ± 0.04	0.09 ± 0.05	0.06 ± 0.03	0.01 ± 0.01	0.01 ± 0.0	0.01 ± 0.0	0.08 ± 0.01	0.03 ± 0.01	0.02 ± 0.01	0.01 ± 0.0	0.01 ± 0.01	0.04 ± 0.02	0.01 ± 0.0	0.01 ± 0.0	0.01 ± 0.0	0.01 ± 0.01	0.01 ± 0.0
39	26.102	2787.91	Maltose-8 TMS		8.41 ± 2.82^a,b^	8.78 ± 4.0^a^	7.87 ± 4.04^a,b^	0.33 ± 0.22^c^	0.94 ± 0.42^c^	0.32 ± 0.11^c^	3.56 ± 0.8^b,c^	0.45 ± 0.18^c^	2.23 ± 0.47^c^	0.56 ± 0.3^c^	0.28 ± 0.3^c^	2.00 ± 1.44^c^	0.09 ± 0.04^c^	0.08 ± 0.03^c^	0.10 ± 0.03^c^	0.06 ± 0.03^c^	0.18 ± 0.05^c^
41	27.059	2877.75	Melibiose-8 TMS		2.12 ± 0.59	1.12 ± 0.32	1.75 ± 0.04	0.04 ± 0.01	0.30 ± 0.14	0.08 ± 0.03	4.01 ± 0.77	0.40 ± 0.11	0.61 ± 0.22	0.17 ± 0.06	0.27 ± 0.24	3.56 ± 1.65	0.08 ± 0.02	0.10 ± 0.04	0.11 ± 0.03	0.09 ± 0.04	0.67 ± 0.14
**Total sugars**					**42.48 ± 11.53**	**80.97 ± 23.89**	**41.37 ± 13.28**	**14.54 ± 8.49**	**10.79 ± 4.54**	**7.57 ± 2.86**	**283.02 ± 53.29**	**290.86 ± 59.88**	**93.12 ± 29.17**	**8.93 ± 2.95**	**26.85 ± 21.76**	**122.50 ± 44.67**	**3.54 ± 1.02**	**3.56 ± 1.20**	**5.68 ± 2.07**	**5.20 ± 2.49**	**4.02 ± 1.12**
10	16.488	1739.91	Ribitol-5 TMS	Sugar alcohols	0.70 ± 0.27	2.16 ± 0.59	0.93 ± 0.17	0.33 ± 0.23	0.17 ± 0.07	0.12 ± 0.05	10.17 ± 1.59	9.85 ± 2.15	1.81 ± 0.83	0.16 ± 0.06	0.73 ± 0.78	3.76 ± 1.55	0.08 ± 0.01	0.06 ± 0.02	0.10 ± 0.03	0.09 ± 0.04	0.07 ± 0.01
12	17.125	1793.92	Sorbitol-5 TMS		0.57 ± 0.14	1.74 ± 0.45	0.68 ± 0.48	0.24 ± 0.19	0.07 ± 0.03	0.05 ± 0.02	5.14 ± 0.43	3.58 ± 1.26	0.95 ± 0.5	0.07 ± 0.04	0.22 ± 0.16	0.92 ± 0.46	0.06 ± 0.04	0.04 ± 0.03	0.07 ± 0.04	0.04 ± 0.02	0.06 ± 0.01
16	17.532	1831.39	Iditol-6 TMS		0.24 ± 0.24	0.29 ± 0.15	0.15 ± 0.14	0.05 ± 0.04	0.08 ± 0.11	0.07 ± 0.08	1.30 ± 0.37	1.14 ± 0.35	0.39 ± 0.13 ± 0.0	0.12 ± 0.08	0.16 ± 0.13	0.34 ± 0.17	0.06 ± 0.03	0.04 ± 0.02	0.04 ± 0.04	0.03 ± 0.01	0.04 ± 0.05
19	17.812	1857.6	1,5-Anhydro-d-glucitol-5 TMS		0.05 ± 0.01	0.09 ± 0.02	0.05 ± 0.03	0.04 ± 0.02	0.02 ± 0.01	0.02 ± 0.01	0.24 ± 0.05	2.74 ± 2.18	0.08 ± 0.03	0.02 ± 0.0	0.03 ± 0.02	0.15 ± 0.04	0.01 ± 0.0	0.00 ± 0.0	0.01 ± 0.0	0.01 ± 0.01	0.01 ± 0.01
20	18.04	1879.48	Pinitol-5 TMS		0.03 ± 0.0	0.04 ± 0.0	0.04 ± 0.0	0.03 ± 0.01	0.02 ± 0.01	0.02 ± 0.0	0.01 ± 0.01	0.01 ± 0.01	0.02 ± 0.0	0.02 ± 0.0	0.01 ± 0.01	0.02 ± 0.0	0.02 ± 0.0	0.02 ± 0.0	0.02 ± 0.0	0.02 ± 0.01	0.02 ± 0.0
21	18.305	1904.17	Unknown-6 TMS		0.12 ± 0.07	0.37 ± 0.20	0.22 ± 0.16	0.08 ± 0.05	0.04 ± 0.01	0.02 ± 0.01	0.47 ± 0.17	0.34 ± 0.13	0.26 ± 0.08	0.03 ± 0.01	0.04 ± 0.03	0.11 ± 0.09	0.01 ± 0.0	0.01 ± 0.0	0.01 ± 0.0	0.01 ± 0.0	0.01 ± 0.0
27	19.483	2016.52	Unknown-5 TMS		0.25 ± 0.06	0.43 ± 0.08	0.24 ± 0.03	0.05 ± 0.02	0.03 ± 0.01	0.04 ± 0.01	1.67 ± 0.22	2.29 ± 0.57	0.42 ± 0.16	0.05 ± 0.01	0.14 ± 0.16	0.61 ± 0.2	0.02 ± 0.0	0.02 ± 0.0	0.03 ± 0.01	0.02 ± 0.01	0.02 ± 0.0
29	20.408	2112.01	Myo-inositol-6 TMS		4.08 ± 0.79	4.34 ± 1.15	4.55 ± 0.97	1.22 ± 0.49	0.93 ± 0.33	1.25 ± 0.22	10.09 ± 1.34	18.15 ± 3.72	5.69 ± 1.07	1.57 ± 0.2	3.75 ± 1.8	6.48 ± 1.79	0.85 ± 0.15 ±	0.84 ± 0.17	0.94 ± 0.1	0.66 ± 0.19	1.00 ± 0.16
**Total sugar alcohols**					**6.04 ± 1.57**	**9.46 ± 2.61**	**6.86 ± 1.98**	**2.03 ± 1.05**	**1.33 ± 0.57**	**1.58 ± 0.40**	**29.10 ± 4.17**	**38.10 ± 10.38**	**9.31 ± 2.79**	**2.04 ± 0.40**	**5.08 ± 3.08**	**12.39 ± 4.29**	**1.11 ± 0.24**	**1.04 ± 0.26**	**1.23 ± 0.24**	**0.88 ± 0.29**	**1.23 ± 0.25**

One-way ANOVA was performed for markers appeared in PCA and OPLS-DA loading and S-loading plot models, respectively. The samples code is described in Table 1**.**

The superscript small letters (a–e) represent the Tukey Pairwise Comparisons between investigated samples for marker metabolites revealed in chemometrics model. The grouping information are based on 95% confidence, where means that do not share a letter are significantly different.

The bold values are the sum of each class of the identified phytochemicals.

### Sugars

Sugars amounted for the most abundant class in kernels represented by mono- and di-saccharide ranging from 3.5 to 290.9 µg/mg among cvs. The highest sugar level was detected in cvs. QQS, QRS, and QSS detected at 290.9, 283, and 122.5 µg/mg, respectively. Moderate levels were likewise detected in QZS (93.1 µg/mg) and SKS specimens (81 µg/mg) compared with QHS and QGS cvs. that showed the least level (3.5 µg/mg) (Table 2). In that context, the comparative study displayed that sugar content was variable within mango kernels that originated from Sharqia province and Suez. This finding was in agreement with previous literature of which sugars do not represent strong taxonomical or geographical markers being influenced by agricultural practices independent of location or cv. type^26^. Noticeably, monosaccharides viz. glucose, fructose, and talose were the major sugars, while disaccharides represented by maltose and melibiose were found at much lower levels in all kernels, and suggestive that mono sugars amounted for the major sugar source in kernels, and in agreement with our previous results in mango fruit^11^. Sucrose, fructose and glucose represent the principal sugars being detected in mature and ripe mango^31,32^, however traces of sucrose were detected in kernels.

With regard to cvs. collected from multiple sites in Egypt, Zabdeya as one of the most popular and highly consumed mango type in Egypt was collected from Suez, Sharqia, and Giza with notable differences in sugar levels (4.0–93.1 µg/mg) likely attributed to agricultural practices. Highest total sugar level was detected in kernels from Sharqia in QZS at 93.1 µg/mg followed by SZS at 42.5 µg/mg, while cvs. collected from Giza GZS showed lowest level at 4.0 µg/mg (Table 2). Such differences in sugar level among kernels collected from different regions was also observed though to less extent in case of Aweis and Fons kernels showing only slight variation in sugar levels based on origin, being detected at 14.6 µg/mg in SAS versus 8.9 µg/mg for QAS, while SFS and QFS encompassed 10.8 and 7.6 µg/mg, respectively.

### Sugar alcohols

Sugar alcohols have increasing attention in dietary nutrition and health as low calorie sweeteners in bakery, beverage and confectionary^33^. These sugars are not readily absorbed providing fewer calories than other table sugars^34^. In mango kernels, sugar alcohols predominated in all cvs. ranging from 1.0 to 38.1 µg/mg, with the highest level found in Sharqia province represented by QQS and QRS cvs. at 38.1 and 29.1 µg/mg. Moderate levels were detected in Suez samples in SKS, SNS, and SZS ranging from 6 to 9.5 µg/mg.

Interestingly, one of the premium mango types including Fons and Aweis cultivated in both Suez and Sharqia recorded almost comparable sugar alcohols´ level at 1.3 and 1.6 µg/mg for SFS and QFS, whilst 2.0 µg/mg for SAS and QAS cvs. In contrast, Zabdeya (SZS, QZS, and GZS) from the three locations; Suez, Sharqia, and Giza, induced a clear variance in sugar alcohols´ level being detected at 6.0, 9.6, and 1.2 µg/mg respectively (Table 2). This finding was consistent with sugar levels suggestive that sugar alcohols could be affected by origin.

With regards to sugar alcohols, 1,5-anhydro-d-glucitol was detected at highest the level 2.7 µg/mg in QQS versus trace levels in all other kernels. It should be noted that few reports have investigated the health impact of 1,5-anhydro-d-glucitol, and little is known about its actions in vivo being a rare saccharide^35^. 1,5-anhydro-d-glucitol showed competitive inhibition of trehalase and trehalose phosphorylase, and is likely attributed to its structural similarity with d-glucose^36^, asides from its antidiabetic action as a low calorie sugar^37^.

Major sugar alcohols detected in kernels included ribitol, iditol, pinitol, and myo-inositol, with myo-inositol as major form detected at 20.3 µg/mg in QQS cv followed by QRS at 11.6 µg/mg, and in accordance with previous report revealing for its richness in mango fruit^38^. Myo-inositol is a potential sugar alcohol as low calorie sugar asides for its role in normal cell growth and survival, development and function of peripheral nerves^39^. The richness of QRS in myo-inositol, was also observed in case of ribitol at 10.1 µg/mg compared to other cvs. (0.1–3.7 µg/mg) (Table 2) and posing this cv. as potential source of sugar alcohols.

### Fatty acids/esters

Fatty acids represented the second most abundant class in kernels as expected with mango fruits being enriched in fats^11^. No major differences were observed in fatty acids profile amongst the selected specimens either based on cv. type or localities. The total fatty acids were identified in all cvs. detected at levels ranging from 14.3 to 18.8 µg/mg, with the highest levels in QSS (18.8 µg/mg) versus lowest in QGS at 14.3 µg/mg (Table 2). Mango peels and kernels are regarded as most rich in lipids presenting good source of fatty acids^13^, some of which have the potential to be exploited in food industries^13^.

With regards to newly reported lipid species in mango kernel, fatty acid ester i.e., butyl caprylate was detected in most cvs. as a major form for the first time ranging from 11.7 to 14.4 µg/mg (Table 2). This compound was previously identified as main volatile constituent in mango aroma profile to display a potential repellent activity against insect pests^40^, adding to fruits shelf life. It also possesses a pleasant flavor and fragrance features posing QSS, SZS, SNS, QAS, and QDS cvs as the most rich source of that natural flavoring agent^41^. Aroma profiling of mango kernel using more sensitive techniques such as SPME should be considered based on these results. In comparison, monoglyceride non-volatile conjugates exemplified by 1-monopalmitin were detected at lower levels in most cvs. at 2–3 µg/mg, versus trace levels of methyl palmitate, palmitic, linoleic, and stearic acids. These results are though not in accordance with previously reported data in which linoleic, stearic, palmitic, and oleic acids were the major fatty acids in mango fruit^13^, and suggestive for different lipid profile in kernels from that of the fruit which has yet to be compared for mango from other origins. It should be noted that the high level of stearic, oleic, and palmitic acids enhanced mango kernel fats to be employed as cocoa butter substitute^6^, and to add to mango kernel fat nutritive and health properties. QRS and QQS cvs were found the richest in these fatty acids.

### Phenolics/phenolic lipids

Phenolics and phenolic lipids are well known as potential antioxidant chemicals in food products asides from several health benefits^42^. The highest phenolics and phenolic lipids levels was detected at 18.3 µg/mg in QQS specimen followed by QZS, QDS and SKS cvs., later detected at 10.8, 10.6, 10.1 µg/mg, respectively. Quantitative differences in phenolics were detected in cvs. from Sharqia province, with highest level found in QQS (18.4 µg/mg) versus lowest in QGS (4.2 µg/mg). Beside Sharqia, Suez province was recorded a change in phenolics in which predominated with greater amount in SKS cv. (10.1 µg/mg) comparing to SNS (1.7 µg/mg) (Table 2). Additionally, comparable levels of phenolics were detected in Aweis kernels collected from different geographical regions (Suez and Sharqia) at ca. 8.4 µg/mg for QAS and SAS, and suggestive that phenolics provide better markers for cvs. than sugars as previously identified. Likewise, comparable levels of total phenolics were detected in Zabdeya and Fons cvs. from different origins i.e., Suez, Sharqia and Giza at 8–10 µg/mg in case of QZS, SZS, and GZS versus 4–6 µg/mg in SFS and QFS, respectively. These results confirm that specialized metabolites present better markers for classification of cvs., not being affected by regional habitat versus primary metabolites such as sugars, and in accordance with our previous results in other food^26^.

On the other side, it should be noted that four phenolic lipids were detected in mango kernels for the first time including phenol (3-heptadecenyl)-(cardanol), phenol(3-heptadecenyl)-(ginkgol), 1-(2,3-dimethoxyphenyl)ethanol, and 3-(eptadecadienyl) phenol. Ginkgol and cardanol were the major components amongst all cvs. ranging from 0.1 to 13.5 µg/mg (Table 2), and likely to contribute to mango kernels shelf life considering their potential antimicrobial actions^43^. In that regard, QQS should be assessed for its antimicrobial action against food borne pathogens considering its rich cardanol content (13.5 µg/mg). Examination of the potential health benefits of these phenolics should now follow to identify functional food or other uses for these mango kernels based on such metabolite profiling results.

### Amino acid/nitrogenous

Amino acids are formed due to various metabolic processes during ripening stages of fruit maturity^44^, and detected in all cvs. at comparable levels ranging from 6.3 to 9.3 µg/mg. The amounts detected in Sharqia ranged from 6.3 to 8.4 µg/mg, and comparable to that in Suez cvs. being detected at 7.8–9.3 µg/mg. Besides, amino acids in Ismailia and Giza collected accessions represented by IMS and GZS were at similar levels of 8.5 and 9.1 µg/mg, respectively. Hence, amino acids content does not appear to be affected by cvs. or origin in this study. Profiling of mango kernels revealed for 5 major amino acids and nitrogenous compounds exemplified by sarcosine, ethyl ester, nicotinic acid and L-threonine, with sarcosine-methyl ester as major components ranging from 3.3 to 4.7 µg/mg. Sarcosine is recently recognized for its CNS effects against depression, anti-inflammation in the brain^45^, in addition for management of schizophrenia and Alzheimer’s disease^46,47^.

### Miscellaneous

In addition to the aforementioned classes, other chemicals were detected though at trace levels including fatty alcohols, ketones and acids. Fatty alcohols were represented by two components including behenic alcohol and 1-docosanol detected at highest level in QRS sample at 2.0 µg/mg while traces of ketones and acids were identified in all examined cvs. Behenic alcohol was the major fatty alcohol in all tested kernels detected at 1.3 µg/mg at presented in Table 2.

### Multivariate data analysis (MVA) of mango kernels in context to its cv. and/or geographical origin

**Figure 3:**
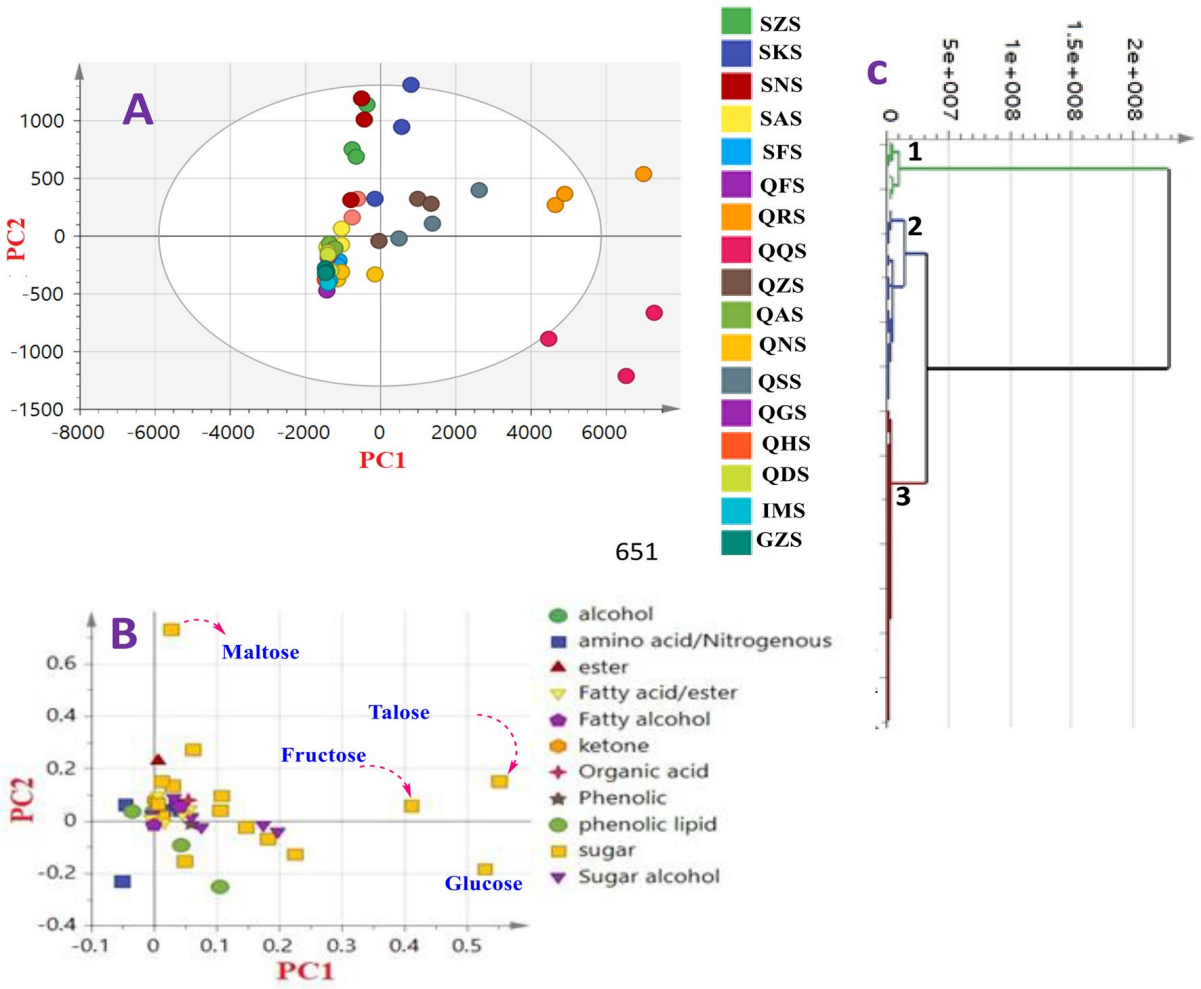
GC–MS based principal analyses of different mango cvs. The metabolome clusters are located at the distinct positions described by two vectors of PC1 (86%) and PC2 (4.3%). **(A)** Score plot of PC1 versus PC2 scores. **(B)** Loading plot for PC1 and PC2 with contributing mass peaks and their assignments. **(C)** HCA dendrogram analysis of mango cultivars based on group average cluster analysis using GC–MS. For mango codes refer to Table 1.

MVA was further employed including principal component analysis (PCA), hierarchical cluster analysis (HCA), and orthogonal partial least square (OPLS) analysis for specimens’ classification in an untargeted manner (Fig. 3).

### Unsupervised PCA and HCA data analysis of different cvs of mango’s kernels

PCA is a potential modelling tool for assigning relative variability within cvs: from different origins, and to assess for method reproducibility^48^. Clustering result indicated that biological replicates for each cv: were clustered together suggestive of low biological variance within each specimen as results of replicates were more or less superimposable. The model described by principal component (PC1) and PC2 accounted for 90% of the total variance (Fig. 3A). PCA score plot indicated that QRS, QSS, and QZS specimens all from Sharqia province were well spaced and positioned on the right side of PC1, with QQS and some of QRS were clearly appearing as outliers. The left side of negative PC1 score plot showed an overlap between the remaining cvs belonging to GZS, SZS, SNS, and IMS. The close clustering of negative side samples of PC1 without significant segregation indicated their similar metabolite profiles in these cvs. Investigation of the loading plot (Fig. 3B) revealed that sugars mostly accounted for cvs segregation. For instance, talose, fructose, glucose and maltose were abundant in cvs in QRS, SKS and QSS cvs.

HCA was implemented for cvs. classification in an intuitive graphical displayed, Fig. 3C. Three clusters were observed in HCA dendrogram, with only Sedeeq cv. from Sharqia province (QQS) clustered in a separated group (group 1), whilst 4 cvs. originating from Suez and Sharqia viz. QZS, QSS, SNS, and SKS were clustered in group 2. The remaining cvs from different localities were all combined in group 3 suggestive of their more or less similar metabolic components, and in agreement with PCA results (Fig. 3A).

### Unsupervised PCA of cvs. based on origin Sharqia versus Suez

**Figure 4:**
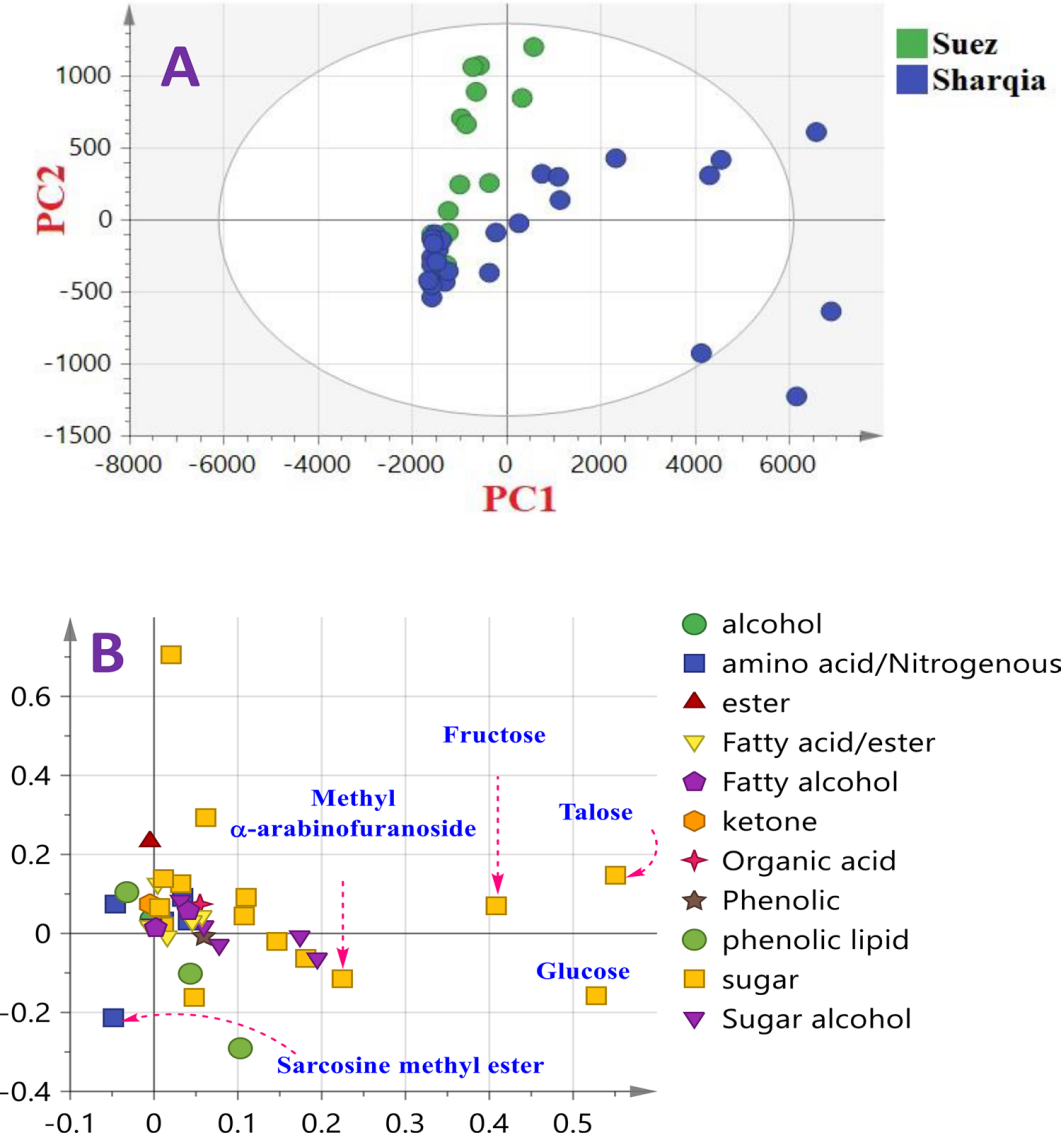
GC–MS based principal component analysis of mango cvs. originated from Suez (green color) and Sharqia (blue color). The metabolome clusters are located at the distinct positions described by two vectors of PC1 and PC2 with total variance 91.1%. **(A)** Score plot of PC1 versus PC2 scores. **(B)** Loading plot for PC1 and PC2 with contributing mass peaks and their assignments. For mango codes refer to Table 1.

We further attempted to assess whether a clear separation among cvs can be observed based on geographical origin. Mango’s kernels were stratified based on locality for origins from Sharqia denoted in blue and Suez in green. Whilst most of cvs. from Suez were segregated in the right side with positive PC1 values, some samples overlapped with kernels from Sharqia on the negative side along PC1 (Fig. 4A) representing 86.7% of the total variance and suggestive that no classification can be readily inferred from kernel derived trees origin from such dataset.

The corresponding loading plot (Fig. 4B) revealed that the samples in the right side were more enriched in sugars viz. fructose, talose and arabinose, whilst sarcosine methyl ester was more detected in cvs present in the left side of PC1.

### PCA of Zabdeya from the three localities

**Figure 5:**
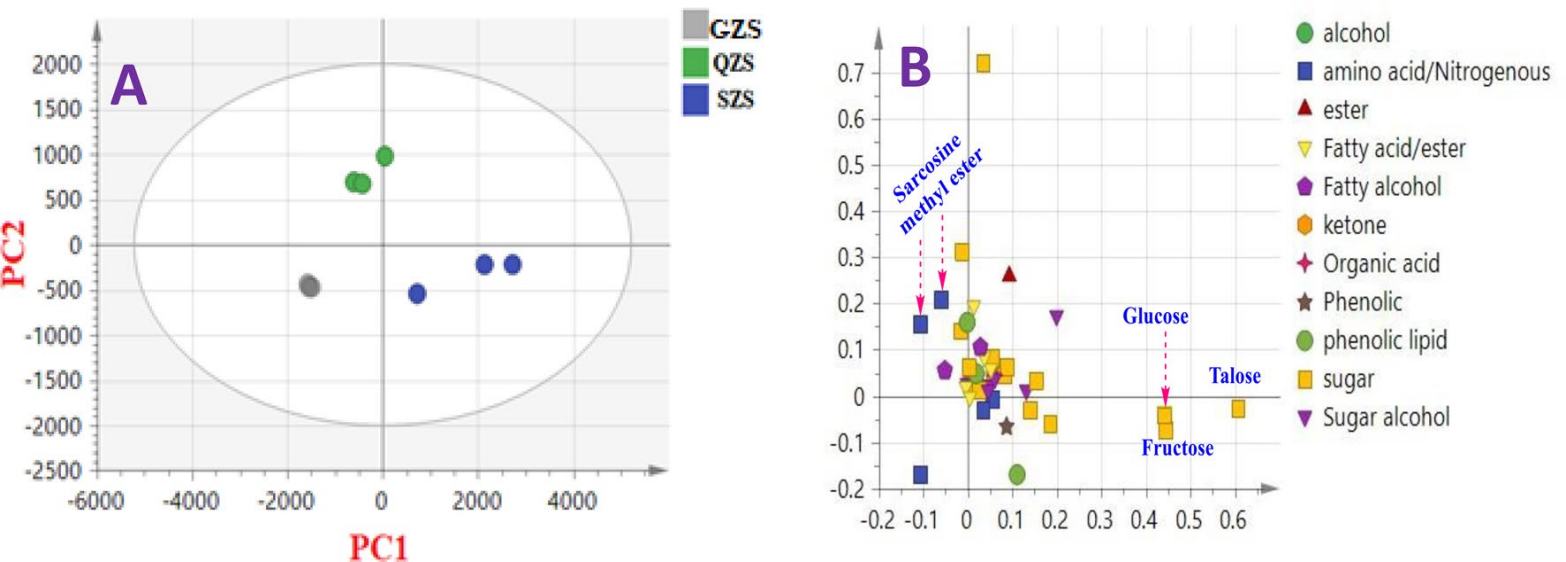
**(A)** Score plot of PC1 (81.6%) versus PC2 (12%) scores of Zebdya kernels from different localities viz. GZS, QZS, and SZS. (**B**) Loading plot of PC1 and PC2 of Zebdya. For mango codes refer to Table 1.

To confirm whether clear distinction for one cv. can be observed from three locations, kernels from Zabdeya cv. being derived from three different localities “Suez, Sharqia and Ismailia” were modelled using unsupervised PCA. Figure 5 shows PCA score (A) and loading (B) plots for Zabdeya cv from the three locations within total variance coverage of 93.6% along PC1 and PC2. Figure 5A revealed acceptable segregation of cv. based on locality. Loading plot (Fig. 5B) showed that Zabdeya from Sharqia richness in sugars, i.e., glucose, talose, and fructose, and in agreement with PCA results (Fig. 3) for kernels from this region richness in sugars. In contrast, sarcosine methyl and ethyl esters predominated in other kernel origins.

### Supervised OPLS of Aweis and Fons against other cvs

**Figure 6:**
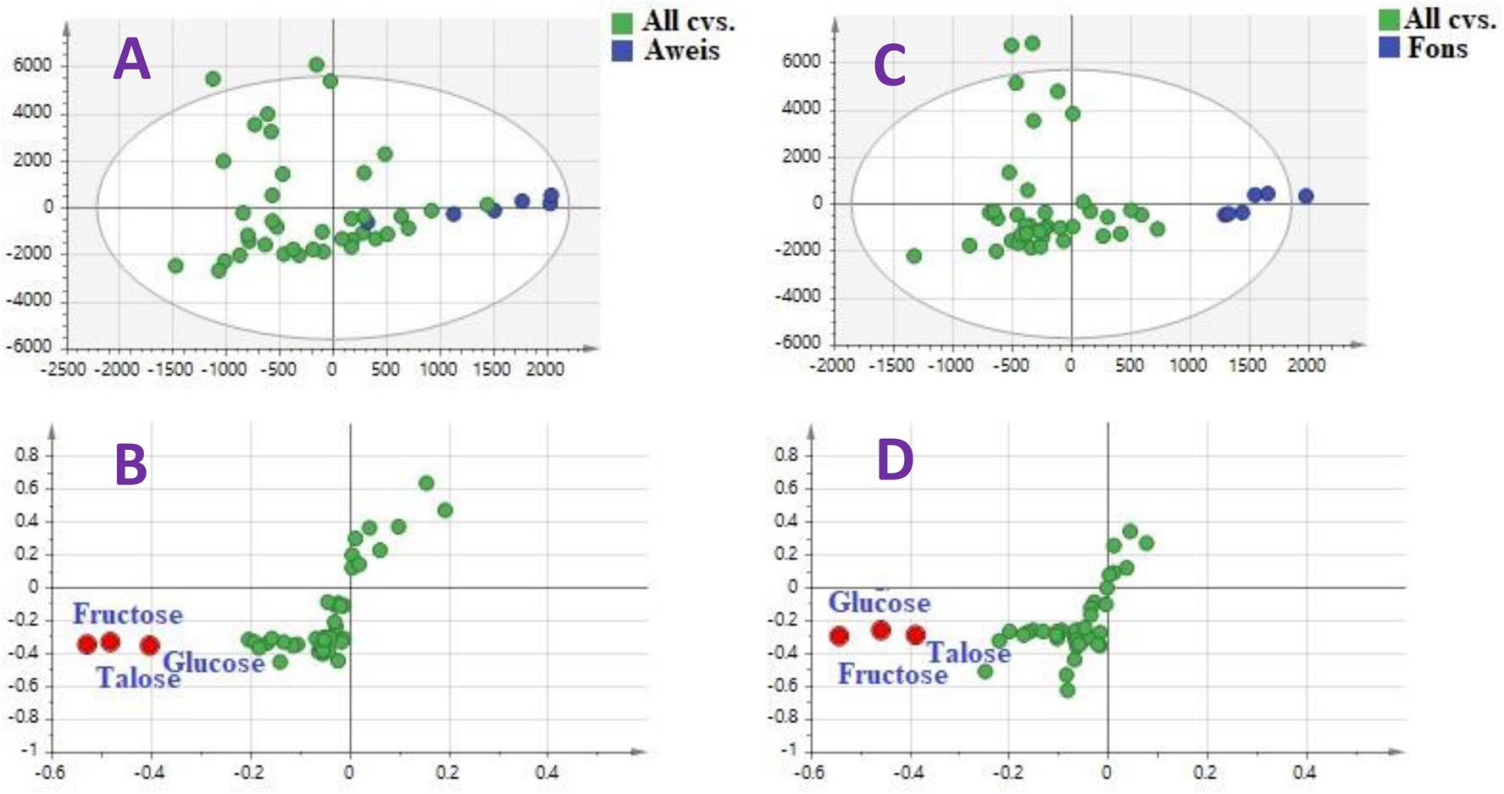
** (A)** OPLS-DA score plot and **(B)** loading S-plots of Aweis mango kernels. **(C)** OPLS-DA score plot and **(D)** loading S-plots of Fons; each modelled one at a time against other cvs.

Considering that both Aweis and Fons are considered premium mango tree types in the Egyptian market regarding their fruit’s composition, we attempted to observe using supervised MVA whether their kernels likewise have unique metabolite profile. Aweis kernels from different origins were modelled as one class group against all other kernel cvs., Fig. 6A with no clear separation of all its kernel specimens from other specimens mostly attributed for its richness in sugars, i.e., fructose, talose, and glucose as revealed from corresponding S loading plot (Fig. 6B). On the other hand, Fons cv. was chemically distinct from other cvs. in which clear separation was observed (Fig. 6C). The S-plot derived from Fons cv. against other mangos revealed that sugars, i.e., fructose, talose, and glucose accounted for discrimination of other cvs. (Fig. 6D). Thus, data analyses confirmed the previous quantification results in Table 2 and showed that the premium mango cvs. "Aweis and Fons" had lower sugar content, particularly glucose, fructose, and talose, enrichment. This may point to the variations in nutrients found in the edible part of the mango fruits, i.e., fruit pulps. It should be noted though that sugars do not present potential markers for cvs. being detected in specimens’ asides from their regulation by several other factors.

## Conclusion

Metabolites heterogeneity in nutrient and volatile profiles of discarded kernel part from 17 mango cvs. originated from three localities alongside Egypt (e.g., Suez, Sharqia, Ismailia, and Giza) is comprehensively investigated herein for the first time via a holistic untargeted GC–MS based volatiles. A total of 41 constituents were identified belonging to sugars, fatty acids/esters, amino acids, and sugar alcohols as the major metabolite classes. Data analysis revealed that cvs. from Sharqia province was recorded a variable sugar content. Amongst other cvs. QQS, QRS, and QSS were the most abundant being detected at 297.1, 288.4 and 123.5 µg/mg. These results were further outlined in case of sugar alcohols data being detected at higher levels in QQS and QRS cvs*.* at 31.7 and 23.7 µg/mg compared with other localities. Major sugar alcohols included ribitol, iditol, pinitol, and myo-inositol, with the ribitol highest level detected in QRS cv. posing it as a potential source of sugar alcohols. In terms of novel chemicals detected in mango kernels, butyl caprylate was detected for the first time at 11.7–14.4 µg/mg. Other newly reported phenolics included ginkgol and cardanol in selected specimens at 0.1–13.5 µg/mg. These phytochemicals play a significant role enhancing mango kernels shelf life due to their potential antimicrobial action. In this regard, QQS should be addressed for assessment of its antimicrobial effect owing for its rich cardanol content (13.5 µg/mg). Therefore, the potential use of mango kernels for food or other applications with regard to the obtained characterized chemicals are highly recommended to be considered in nutraceutical preparations and as, for example, substitute of sugar cane and food preservative. MVA data confirmed quantification results and displayed that the premium mango cvs. “Aweis and Fons” were less enriched in sugars such as glucose, fructose, and talose. This might indicate the nutritional differences with the fruit pulps. Such comprehensive metabolite profiling could assist in identifying the best cvs. enriched in a certain chemical for future valorization purposes, and likewise targeting specialized metabolites to be analysed using LC–MS more suited for profiling such class of metabolites.

### Supplementary Information

Supplementary Figures.

## Data Availability

The datasets used and analysed during the current study would be available from the corresponding author on reasonable request.
