# Supplementary material for: Valorization potential of Egyptian mango kernel waste product as analyzed via GC/MS metabolites profiling from different cultivars and geographical origins
Source: Sci Rep. 2024 Feb 5;14:2886. doi: 10.1038/s41598-024-53379-4 (PMC10838926; doi:10.1038/s41598-024-53379-4)
Supplement: Supplementary file 1 — Supplementary Figures. [file 41598_2024_53379_MOESM1_ESM.docx]

**Suppl figure**

**Figure S1.** Map of Egypt showing the tree location. Specimens were coded according to the cv. type and geographical origin as explained in **Table 1**.

**
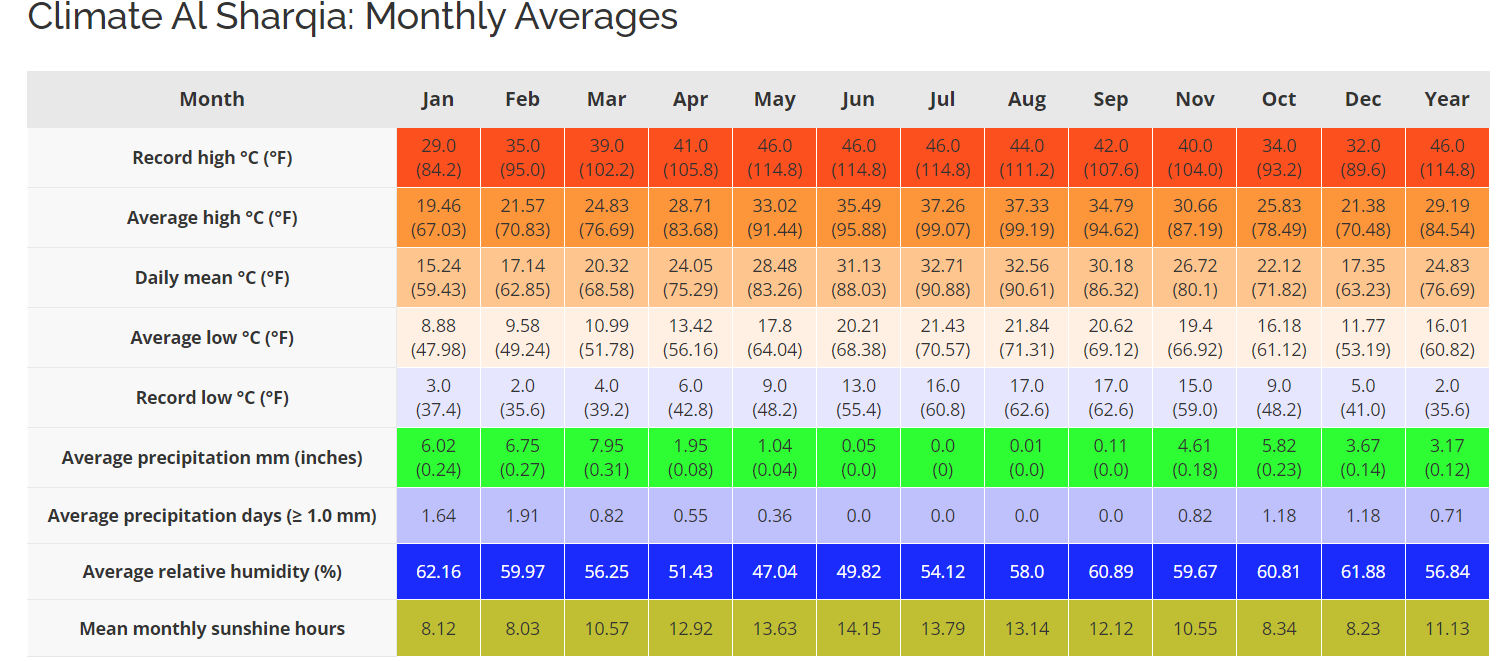
**

**
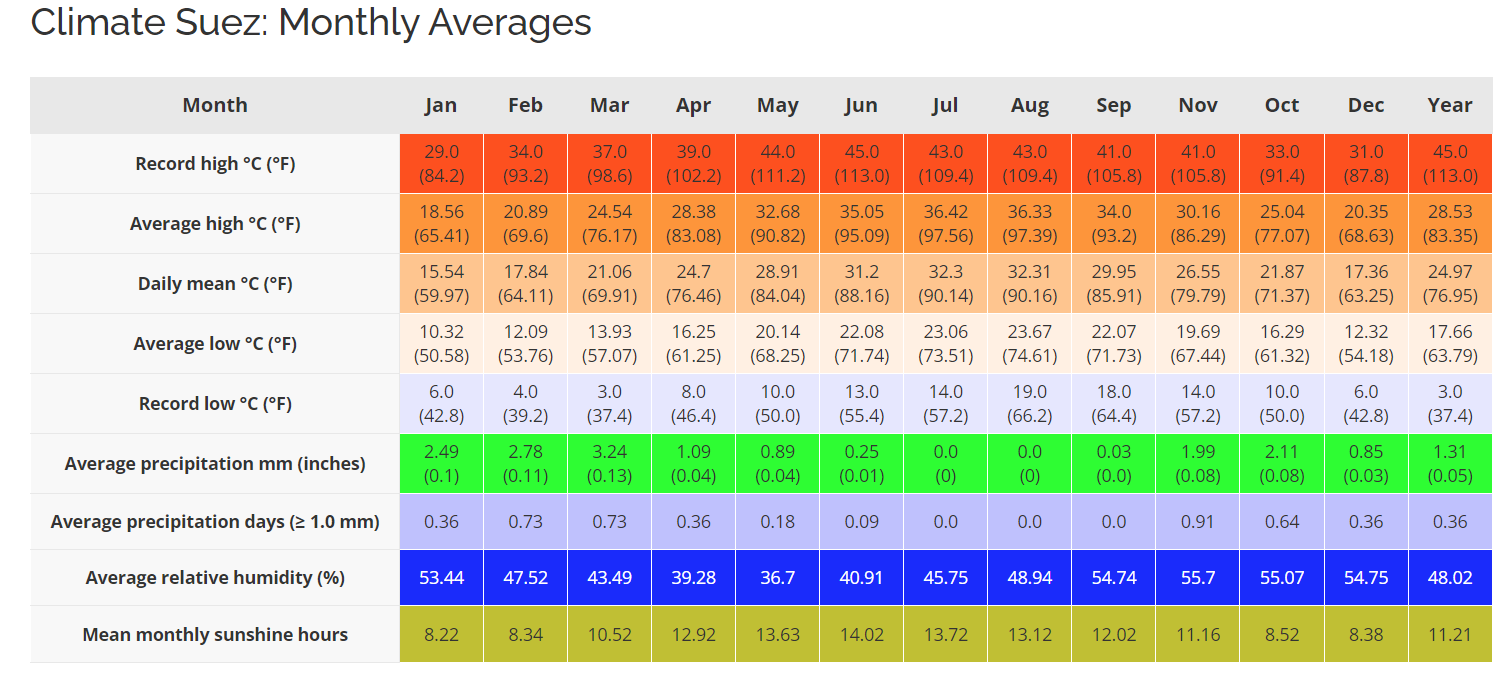
**

**
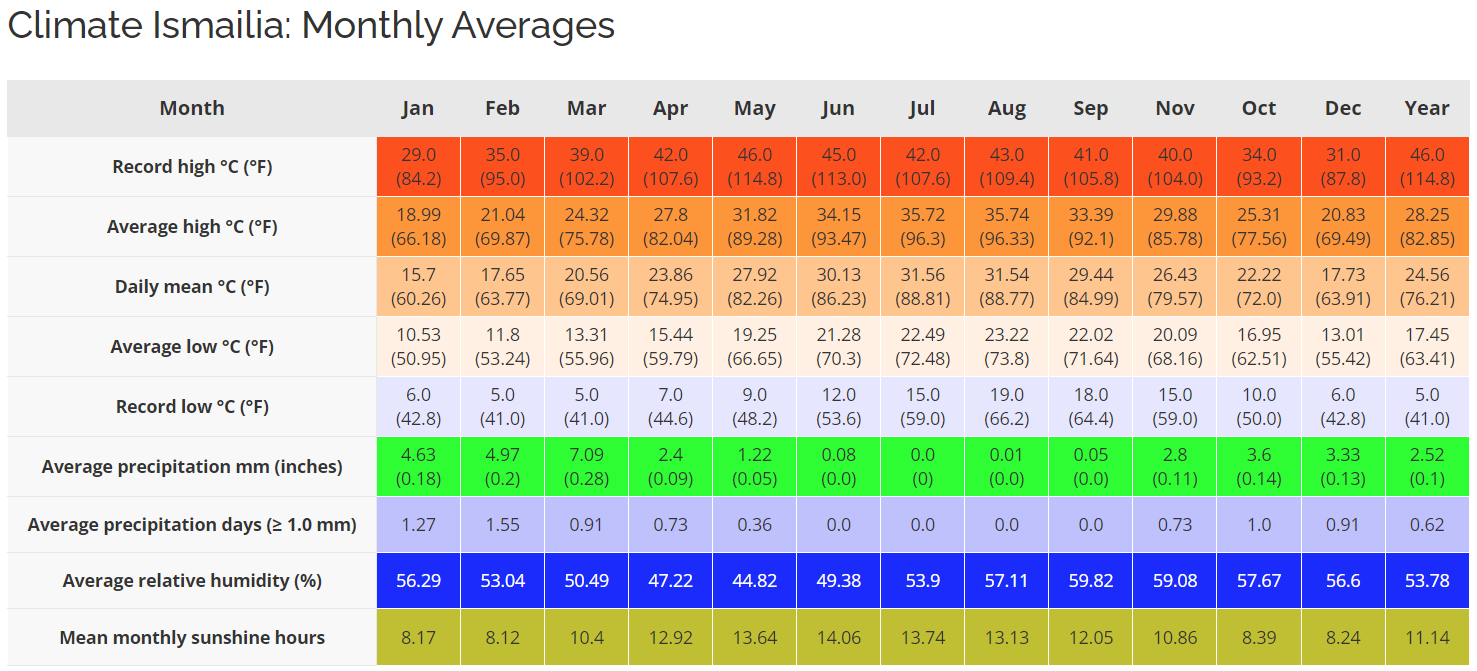
**

**Figure S2.** Climatic conditions of the three different localities alongside Egypt (Suez, Sharqia and Giza). (**Source:** <https://weatherandclimate.com>, data accessed on 13 Jan. 2024).
